# Migrant Home Care Workers in the UK: a Scoping Review of Outcomes and Sustainability and Implications in the Context of Brexit

**DOI:** 10.1007/s12134-021-00807-3

**Published:** 2021-03-29

**Authors:** Agnes Turnpenny, Shereen Hussein

**Affiliations:** 1 PSSRU, University of Kent, Canterbury, UK; 2 London School of Hygiene and Tropical Medicine, London, UK

**Keywords:** Migrant care workers, Social care, Scoping review, Sustainability, Brexit, European Union

## Abstract

Migrant care workers play a significant role in meeting the escalating demand for social care in the UK. Workforce shortages create opportunities for new migrants to enter the social care workforce. This scoping review aims to identify and synthesise available evidence on the contribution of migrant workers to the provision of home care in the UK focusing on care worker and service outcomes as well as sustainability, and identify challenges and gaps in the context of Brexit and changing immigration policies. Twenty-two articles were identified for inclusion in the review and extracted using a structured format. The analysis presents a narrative description and synthesis of the research. Findings from the reviewed articles were grouped into five main themes: migrant, user and employer outcomes, effect on workforce, and sustainability—and 15 sub-themes that were described in detail. Much of the existing research on migrant care work is qualitative and focuses on migrant outcomes. The review identified some important gaps in research, namely, the impact of immigration status on migrant care worker outcomes, the cultural and psychological adaptation of migrant care workers to care practices, and the emerging UK live-in care market. Implications of findings are discussed in the context of post-Brexit immigration system.

## Background

Migrant workers are increasingly filling labour gaps in long-term care across the world, driven by demographic changes and responding to major challenges in the delivery and cost of care (Anderson, 2012). Although migrant care workers are present in most countries, their share and roles vary significantly and are affected by different factors. Major among these are the welfare state and immigration regimes of host countries (van Hooren, 2012). Demand for migrant care workers is shaped by the institutional structure and specific configurations of the social care system (Anttonen et al., 2003, p. 13; Simonazzi, 2008; Lyon & Glucksmann, 2008). Williams (2012) argues that variations in the employment of migrant care workers are influenced by how employment, migration, and social care systems interact. These systems alone and in interaction also create or contribute to the vulnerability of migrant care workers.

The UK is similar to many European Union (EU) and OECD countries in that it relies heavily on migrant care workers, who are over-represented in the long-term care sector (Cangiano, 2014). In 2020 (prior to the COVID-19 pandemic), non-UK nationals represented 17 per cent of the social care workforce in England (Skills for Care, 2020), compared to approximately 11 per cent of all people in employment (ONS EMP06).

In 2016, the UK voted to leave the EU and as part of the Brexit process, to ‘take back control’ of its borders and ‘end free movement and the preferential treatment’ of EU migrants.^1^ The Brexit decision was followed by years of political uncertainty about the shape and nature the UK’s future relationship with the EU. In 2020, the UK left the EU with a transition period until the end of the year. Following its election manifesto in 2019 (Conservative and Unionist Party, 2019, p. 20), the government will introduce a new immigration system in 2021 that ends rights to free movement for EU workers and implements a points-based immigration system for everyone moving to the UK for work (Home Office, 2020).

Under the new immigration system, few—if any—care worker jobs will allow applicants to collect the required number of points to qualify for a work visa: the majority of positions in social care are below the minimum qualification level. The impact of the new rules on social care are not yet evident, however are likely to be significant in the broader context of high vacancies and turnover in the sector and pressures created by the COVID-19 pandemic (Dromey & Hochlaf, 2018; MAC 2018; Morris, 2020; Skills for Care, 2020).

The aim of this review is to scope and present the available research on the migrant care workforce in one of the segments of the UK social care sector—home care. The analysis will focus on key worker and user outcomes and contribute to the assessment of challenges in the context of Brexit and changing immigration rules.

### Social Care in the UK

Social care in the UK covers personal care and practical support for people who need care and support due to disability, illness, old age, and their carers (NAO, 2018). The two main components are residential care (i.e. care homes and nursing homes) and domiciliary care (care and support provided in one’s home). Social care is a devolved responsibility of the four national governments—England, Scotland, Wales, and Northern Ireland (NI)—that have taken diverging policy directions with funding and individual outcomes (wellbeing) at the centre of reforms over recent decades (Hall et al., 2020).

Pressures on workers’ wages and rights have had a negative effect on the sector’s ability to attract or retain workers (Hudson, 2019). Vacancy and turnover rates have been persistently high (Hussein et al., 2016).

These trends have been set in a broader context of ‘personalisation’ and ‘consumer choice’ primarily delivered via cash for care policies (such as personal budgets and direct payments) in quasi-markets of suppliers of care services (Needham, 2011; Owens et al., 2017). Marketisation and personalisation have created a complex and fragmented landscape of services, particularly in England, where there were 18,200 organisations and 38,000 establishments (e.g. care homes) involved in the provision of adult social care in 2019/2020, ranging from micro-providers to large corporate chains (Skills for Care, 2020). While the majority of social care services are publicly funded and purchased by local authorities via competitive tendering, it is estimated that approximately 35–40% of service users self-fund their own care by sourcing services from an open market (Hall et al., 2020).

Domiciliary or home care represents nearly half of social care jobs and workforce, and it has some distinct features:

- High number of ‘direct care’ jobs and fewer roles requiring formal qualifications. Over half of the domiciliary care workforce in England have no care related qualifications (higher than any other social care sector).
- Insecure and precarious employment practices: Nearly half of the home care workforce in England are on a zero-hour contract, highest rate within the social care sector. Zero-hour contracts are a source of additional stress for home care workers (Ravalier et al., 2019).
- Highest vacancy and turnover rates within the sector. In England, the vacancy rate in domiciliary care was 10% in 2018/2019 compared to 7.8% for the sector as a whole or 5.9% in residential care.
- Live-in care is a small but significant segment of the home care market in the UK. It relies heavily on migrant care workers, often recruited directly from abroad.
- Home care workers are significantly more likely to experience ‘high strain’ jobs than their colleagues working in residential care (Hussein, 2018).
- Care is delivered in clients’ homes, and home care workers often work alone and thus have less organisational and peer support than those working in residential settings. The relational aspects of home care are also different from institutional forms of care (Denton et al., 2002).
- The rapid spread of “disruptive technologies” such as introductory agencies and “uber-style” technology platforms that connect people who use services directly to those who provide them and its implications for worker and user rights and liability (Bolton & Townson, 2018).

### Social Care Workforce and Migrant Care Workers

The social care workforce is predominantly female (over 80% in England and Scotland) and one of the lowest paid in the UK economy with average pay close to the statutory minimum wage (Skills for Care, 2020). Non-UK nationals make up 17 per cent of the social care workforce as a whole and 15 per cent of the home care workforce in England (no data are available for the other UK nations), with very little change since 2012 (Skills for Care, 2020).

Migrant workers have traditionally contributed significantly to health and social care in the UK (Simpson et al., 2010). The share of EU migrants working in social care has increased since the accession of new Member States from Eastern Europe in 2004 and 2007 and the 2008 immigration reform that introduced restrictions on the direct recruitment of non-EEA workers to ‘low-skilled’ occupations. Currently eight per cent of non-British care workers are EEA, and nine per cent are non-EEA nationals, with significant regional variation (Skills for Care 2019). In 2017, the UK was the second largest destination of ‘mobile personal care workers’ within the EU, receiving a fifth of the intra-EU mobility (Fries-Tersch et al., 2018). By 2018, Romanian and Polish nationals were the two largest groups of workers in social care, followed by nationals from the Philippines, Nigeria, India, and Zimbabwe (Skills for Care 2019). Thus, migrants constitute a significant part of the formal care workforce and offer an additional recruitment pool from which providers in the sector have been filling labour gaps (Cangiano, 2014).

Reliance on migrant workers in the UK is among the highest in the EU, and it has been referred to as ‘a structural feature of the [UK] care sector’ (Cangiano et al., 2009; p. 163). This has also been described as the ‘migrant in the market’ model, where reliance on migrant workers is largely attributed to their willingness to accept—at least temporarily—the low pay and difficult working conditions characteristic of a privatised and residual social care system in exchange for a relatively easy entry to the local labour market (van Hooren, 2012; da Roit & Weicht, 2013). All else being equal, non-UK nationals were more likely to leave a social care job than their British national colleagues (Vadean et al. forthcoming). An important feature of the UK migrant care workforce, currently, is that the majority are recruited locally and have unrestricted right to work in the country (Cangiano et al., 2009, p. 66-67), and they participate in formalised care provision and labour market.

## Aims and Review Questions

This scoping review is part of a larger study investigating migrant labour in the UK home care sector. The review undertaken aimed to take stock of available evidence on the contribution of migrant workers to the provision of home care in the UK and contribute to the assessment of challenges in the context of Brexit and changing immigration rules. In particular, the review set out to examine the following questions:

- What are the experiences of migrant care workers in the UK (migrant outcomes)? What is known about the wellbeing of migrant care workers?
- What drives the recruitment of migrant care workers and what are the actual and perceived benefits of employing them from the perspective of employers (employer outcomes)?
- What are the experiences of care users in relation to home care provided by migrant workers (service user outcomes)?
- What is the impact of migrant workers on the workforce as a whole (workforce outcomes)?
- What is known about the sustainability of supply and demand for migrant care workers (sustainability)?

## Methods

The review used systematic searches and developed a narrative synthesis of the literature.

Studies that comment on any type or aspect of home care provided by migrant care workers in the UK, including comparative studies, were considered for inclusion in the review. Given our focus on current patterns and challenges in the context of Brexit—the end of free movement—only literature published between 2004 and 2019 were included. The year was selected to coincide with the EU’s first Eastward enlargement and the opening up of the labour market to a large number of workers from the new member states (Pollard et al., 2008).

Peer-reviewed publications, PhD dissertations, and reports published as grey literature were eligible for inclusion if they included some empirical analysis of primary or secondary data. Non-empirical studies (e.g. commentaries) and books were excluded. Systematic reviews were screened for potentially eligible studies. Only research published in English was included in the review.

Eight electronic databases were searched using both free text and keyword terms combined with Boolean operators AND and OR (see Table 1). No geographical filters were used to ensure that UK literature was fully captured in the search results. Language and publication date filters were applied to search results.

**Table 1:** Databases and search terms used

Databases	Free text search terms	Keywords
EBSCO (Academic Search Complete, Abstracts in Social Gerontology, CINAHL); SCOPUS; OVID (Social Policy and Practice, PsychINFO, PsychArticles, Embase); ProQuest (Social Science Premium Collection; Dissertations & Theses Global) Web of Science; Pubmed; Opengrey.eu	Migrant*, foreign*, immigrant*, “home care”, “domiciliary care”, “live-in care”, “palliative care”, “elderly care”, “long-term care”, “home nursing”, community care”, caregiving, “social care”, “labo*r migration”	transients and migrants, emigrants and immigrants, home care services, migrant worker, community care, caregiving, long-term care, social care

Titles and abstracts of studies identified through electronic searching were screened against the eligibility criteria by AT, and decisions were reviewed by SH. Original searches were conducted in May–June 2018 and updated in May 2019 and August 2020, and this resulted in no further studies.

A form (in Microsoft Excel) with the following categories was used to extract information from the studies: study aims, review themes, participants, methods, theoretical framework, findings, implications for Brexit, and limitations.

The quality of studies was not assessed in a formal way, and no papers were excluded from the analysis on the basis of risk of bias (Arksey & O’Malley, 2005). However, in the process of data extraction, limitations and potential sources of bias were noted for each paper. The quality of research and the limitations of the evidence base are discussed in more detail in the findings section.

The analysis presents a narrative description and synthesis of the existing literature using the review questions as a conceptual framework. Studies were coded according to their relevance to each of the key topics of the review (i.e. review questions), and data was extracted into the template. This was then analysed using a framework approach (Thomas et al., 2012; pp. 191-93).

## Results

### Search Results

**Fig. 1:**
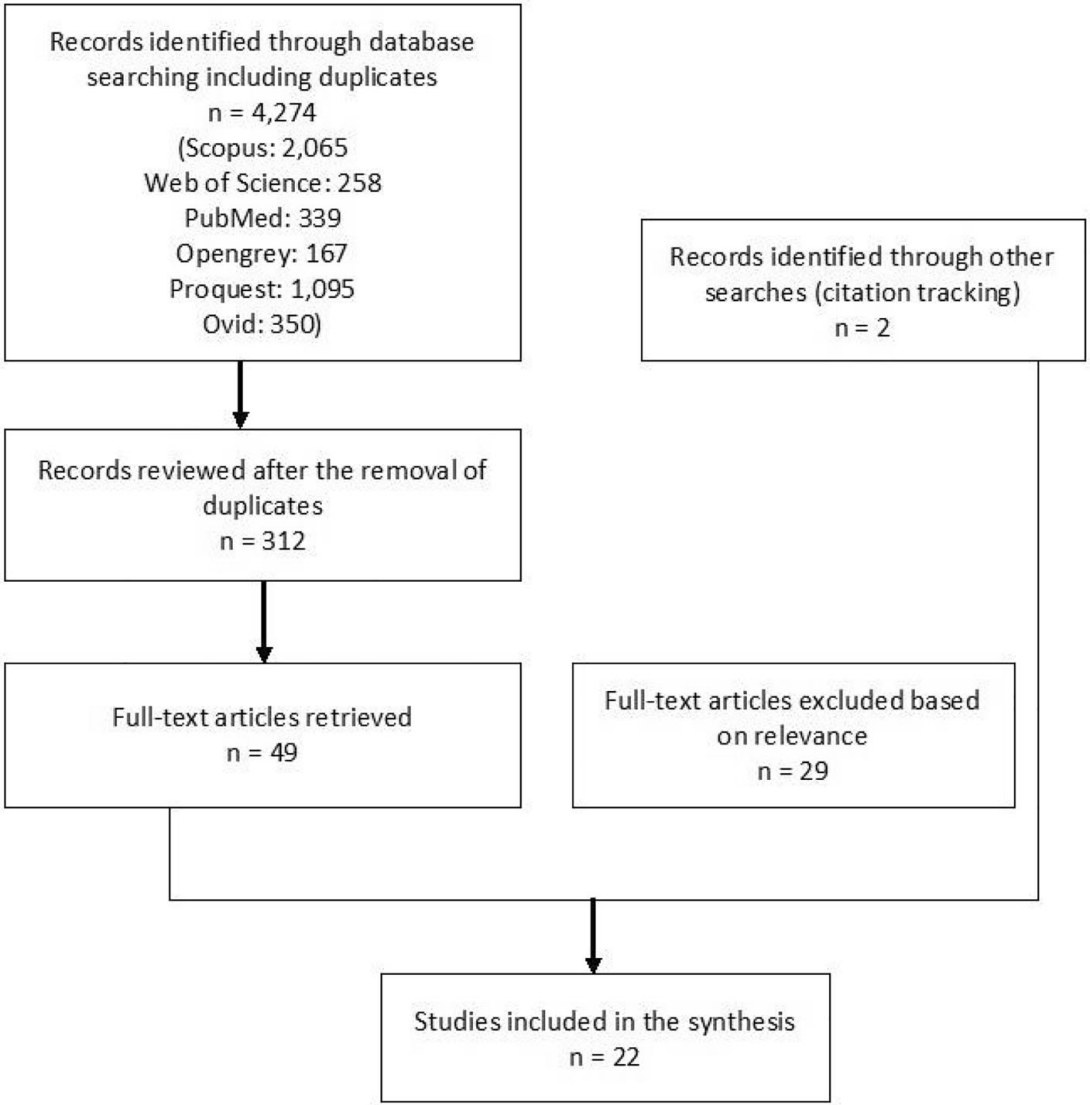
Prisma flow chart

The search of electronic databases yielded 4274 titles, before removal of duplicates across databases. After the removal of duplicates, initial screening, and application of eligibility criteria based on the abstract, 49 papers were marked as potentially relevant (Fig. 1). If a study was available as both a report and a peer-reviewed publication, only the published peer-reviewed paper was included in the review.

Of the 49 studies retrieved, 28 were excluded due to eligibility: lack of empirical analysis or non-UK focus. This left 21 studies for full-text screening and data extraction. At this stage, another study was excluded because it did not include any home care workers, and two further papers were identified through citation tracking. This resulted in a total of 22 studies included in the review (Table 2).

**Table 2:** Studies in included in the review

Authors	Year	Review themes	Area	Aims	Participants and methods
Cangiano & Shutes	2010	Employer outcomes; sustainability; MCW outcomes	Social care	Explore demand for and experiences of migrant care work in the UK	Analysis of demographic and labour force data (LFS), survey of providers (*n*=557), semi-structured interviews with MCWs (*n*=56), focus groups with older people (*n*=5)
Christensen	2017	MCW outcomes	Home care	Examine migrant care worker life trajectories in Norway and the UK	Life story interviews with MCWs (*n*=51; 31 in England, 20 in Norway)
Christensen & Manthorpe	2016	MCW outcomes	Home care	Develop a theoretical understanding of the differentiated risks related to personalised care	Life story interviews with MCWs (*n*=31)
Christensen, Hussein, Ismail	2017	MCW outcomes; sustainability	Social care	Examine patterns of reliance on MCWs from CEE and compare decision-making processes of European migrants joining the LTC sectors in the UK and Norway	Analysis of NMDS-SC records from England and national statistics from Norway; secondary analysis of MCW survey and interview data
Datta et al.	2010	MCW outcomes	Home care	Explore the emergence of a ‘migrant ethic of care’ and how this is shaped by the caring work that migrant women and men do	Questionnaire (*n*=59) and in-depth narrative interviews (*n*=19) conducted in various languages
Hussein & Christensen	2017	MCW outcomes	Social care	Explore why and how migrant men enter low-status social care work (largely seen as ‘women's work’)	Analysis of NMDS-SC records and secondary analysis of previously collected interview data
Hussein, Manthorpe, & Stevens	2011a	Sustainability	Social care	Explore the potential of refugees and asylum seekers in adult social care	Semi-structured interviews with asylum seekers and refugees (*n*=20) and leaders of organisations offering support to refugees (*n*=5)
Hussein, Manthorpe, & Stevens	2011b	MCW outcomes; sustainability	Social care	Test hypothesised relationships between migrant workers’ characteristics, circumstances, experiences and future plans	On-line survey (*n*=101) and focus group. Appr. 25% home care workers. Also includes social workers
Hussein, Manthorpe, & Stevens	2011c	Workforce outcomes; sustainability	Social care	Compare the profile of recent migrant care workers to non-migrant workers in social care	Analysis of NMDS-SC data relating to recent migrant workers whose first job in England was in social care (*n*=5118)
Hussein, Stevens, & Manthorpe	2011	Sustainability	Social care	Understand the drivers for recruiting MCWs	Semi-structured interviews with service users, carers, HR managers, employers, recruitment agencies; UK-born frontline staff, policy stakeholders (*n*=136)
Hussein, Stevens, & Manthorpe	2013	MCW outcomes; sustainability	Social care	Investigate the relationship between stated personal motivations and applicable immigration rules in the UK (i.e. EEA and non-EEA nationals)	Semi-structured interviews with MCWs (*n*=96; 43% Commonwealth, 24% EEA, 33% other countries)
Independent Age / ILC	2016	Sustainability	Social care	Examine the impact of Brexit on the social care workforce and the supply of migrant carers	Analysis of skills for are and ONS data
Manthorpe, Harris, Stevens, & Moriarty	2018	Employer outcomes; sustainability	Social care	Explore the impact of immigration policies on the risk work undertaken by managers who make decisions about the recruitment and employment of migrant care workers	Semi-structured interviews with managers in two cycles: *n*=71 in cycle 1 (2009–2012) and *n*=50 in cycle 2 (2011–2014)
Manthorpe, Hussein, & Stevens	2012	Service user outcomes	Social care	Explore the impact of care provided by migrants on users of social care and their families	Semi-structured interviews with service users (*n*=27) and family carers (*n*=8)
Manthorpe, Hussein, Stevens, & Moriarty	2012	Service user outcomes	Social care	Develop a typology of user preferences and satisfaction with care workers and satisfaction with care	Semi-structured interviews (*n*=35) with service users (*n*=28) and carers (*n*=7)
McGregor	2007	MCW outcomes	Social care	Explore the experiences of highly educated Zimbabweans who left their country in the midst of a political and economic crisis and took up work in social care in England	Semi-structured interviews with Zimbabweans who work in social care (*n*=32)
Read & Fenge	2018	Sustainability	Social care	Examine how social care managers perceive the potential impact of Brexit on future staff recruitment and retention within their organisations	Semi-structured interviews (*n*=5) and questionnaire (*n*=17) with care home and home care managers
Shutes	2012	Sustainability; workforce outcomes	Social care	Examine the ways in which immigration controls shape choice and control by MCWs over their labour	Semi-structured interviews (*n*=56) with three categories of MCWs: unrestricted right to work; restricted rights to work; no right to work
Shutes & Chiatti	2012	Employer outcomes; MCW outcomes; workforce outcomes; sustainability	Social care	Examine how immigration policies and processes shape the employment of migrant workers in the familial provision of care for older people and in the provision of care services in the context of marketisation in England and Italy	UK: Survey sent to a random sample of 3800 care home providers and 500 home care providers (response rate of 12%; *n*=557 (479+78). Semi-structured interviews with 30 providers and 56 MCWs. Italy: Secondary analysis of survey and interview data with migrant care workers and family carers
Shutes & Walsh	2012	Employer outcomes; MCW outcomes; sustainability	Social care	Examine the ways in which divisions of race, ethnicity, and citizenship, as well as the context of quasi-markets in LTC shape the preferences of service providers and service users as regards who provides care, and tensions with MCW’s right to non-discrimination in England and Ireland	UK: Survey sent to a random sample of 3800 care home providers and 500 home care providers (response rate of 12%; *n*=557 (479+78). Semi-structured interviews with 30 providers and 56 MCWs. Five focus groups (*n*=30) with current and prospective service users. Ireland: Survey sent to all 530 care homes and 40 home care organisations (response rate of 50%; *n*=286). Semi-structured interviews with 17 providers and 34 MCWs
Stevens, Hussein, & Manthorpe	2012	MCW outcomes	Social care	To explore the experiences of discrimination and racism, including forms of racism, among MCWs. Part of a larger study (Hussein et al., 2011)	Semi-structured interviews with MCWs (*n*=96; 43% Commonwealth, 24% EEA, 33% other countries)
Walsh & Shutes	2013	Employer outcomes; service user outcomes; MCW outcomes	Social care	Examine the relational aspects of care involving migrant care workers and older people and the implications of these for quality of care in the UK and Ireland	See Shutes & Walsh, 2012

### Description of the Literature

The 22 papers in the review came from a total of seven studies. Four studies contributed more than one publication:

- International social care workers: initial outcomes, workforce experiences, and future expectations, 2007-10 (NIHR-CCF 056/0013) (Hussein, Manthorpe, Stevens)
- Longitudinal Care Work Study, 2009-18 (DH/035/0095) (Hussein, Manthorpe, Stevens, Moriarty)
- The Role of Migrant Care Workers in Ageing Societies, 2007-09 (The Atlantic Philanthropies and Nuffield Foundation) (Shutes, Walsh, Cangiano)
- Welfare policy and care work. A cross-national Norwegian / UK social study of migrants in care work, 2011-12, (Meltzer Foundation, Norway) (Christensen, Manthorpe, Hussein)

Only three papers focused specifically on home care (Datta et al., 2010; Christensen & Manthorpe, 2016; Christensen, 2017). The others included both home and residential care, and one also included social workers. Two studies (McGregor, 2007; Datta et al., 2010) focused on migrants from a specific region (Africa); Hussein et al. (2011a) considered the experiences of care workers who came to the UK as refugees and asylum seekers, and one paper (Read & Fenge, 2019) explored the perspectives of employers on workforce challenges associated with Brexit. All other papers had a broad focus on migrants from any countries, although some commented on similarities and differences between various groups (e.g. EEA, Commonwealth, and non-EEA).

Most papers (*n*=13) were qualitative; there was one quantitative analysis, and eight studies adopted a mixed methods approach combining survey/secondary analysis with qualitative methods.

**Fig. 2:**
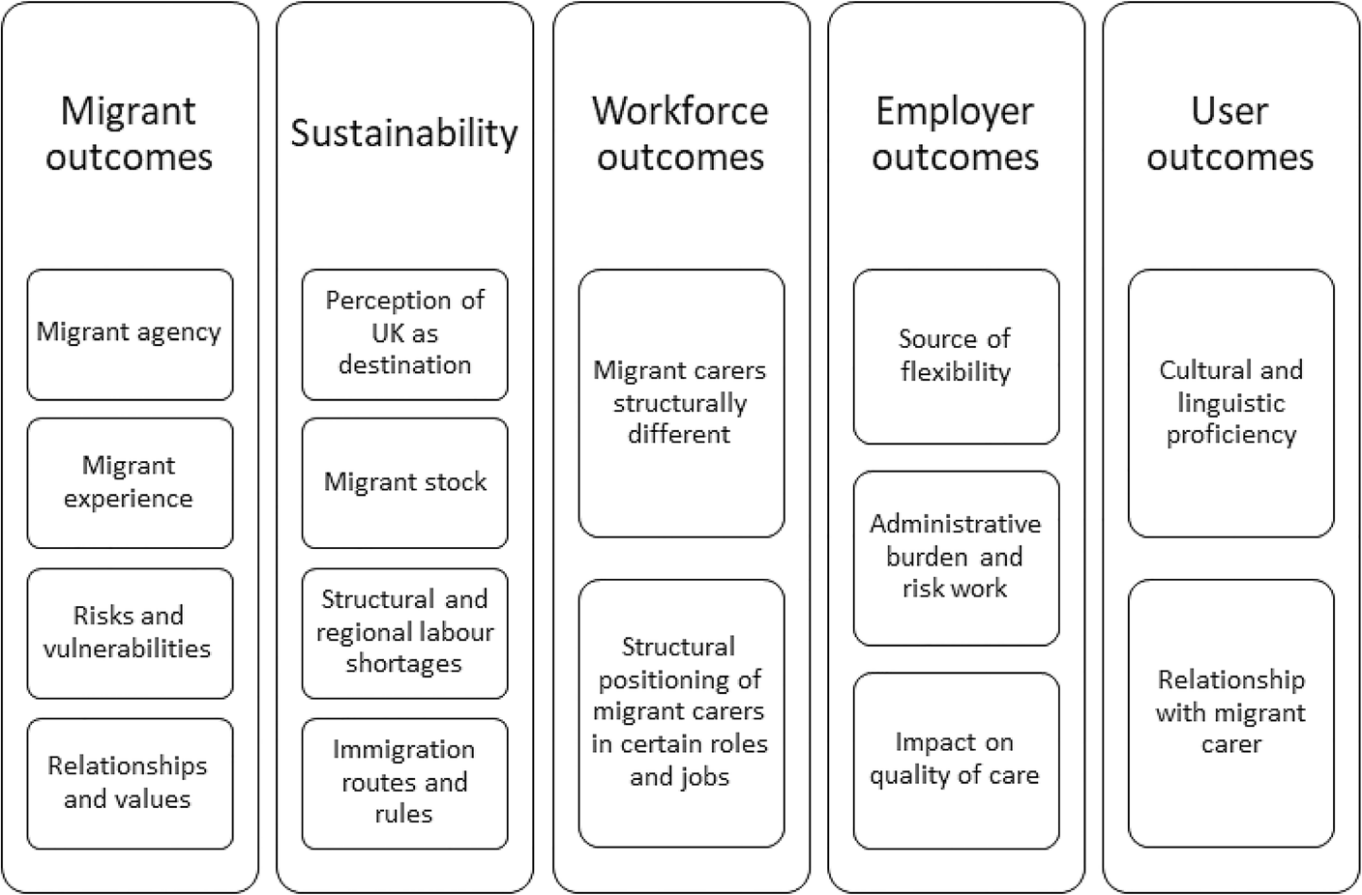
Themes identified in the literature

Most papers addressed more than one topic, and the highest number of studies contributed to the questions of migrant care worker (MCW) outcomes (*n* = 12) and sustainability (*n* = 9), while relatively few studies explored employer (*n* = 4), workforce (*n* = 3), or service user outcomes (*n* = 3) (Fig. 2). The characteristics of studies are summarised in Table 2.

### Synthesis of the Literature

#### Migrant Care Worker Outcomes

Four themes were identified in relation to migrant care worker outcomes: migrant agency, migrant experience, risks and vulnerabilities, and relationships and values in care.

The relevance of ‘migrant agency’ is highlighted in several papers (Christensen, 2017; Christensen et al., 2017; Hussein et al., 2013). The decision to move to the UK and taking up employment in social care are separate steps in the migration trajectory. The UK is often chosen as a direct destination (Christensen, 2017), and the status of the English language as an ‘exportable asset’ potentially facilitates short-term and temporary migration (Christensen et al., 2017). The decision to work in social care is often pragmatic and instrumental (Hussein et al., 2011b). Although migrants recognise the disadvantages of working in social care—low pay, low status, and precarious conditions—they also see it as a stepping stone towards other jobs in the sector or beyond. Researching the Zimbabwean community in the UK, McGregor (2007) shows how becoming a care worker is part of the arrival narrative, and although entry to care often occurs for pragmatic reasons, a number of positive comparisons are made with other sectors that employ large numbers of migrants (such as construction).

Migrant care workers represent a very diverse group in the UK. Christensen et al. (2017) found no dominant settlement tradition in life stories of migrant care workers: some settle and acquire British citizenship but maintain a strong sense of belonging, while others return or move to other countries.

Crossing more traditional gender divisions of labour is another aspect of migrant agency. Migrant men are overrepresented in care work, and their entry and settlement dynamics are also distinct from those of women. Hussein and Christensen (2017) identify three such dynamics: ‘stumbling upon care work’; negotiating a ‘trapdoor’ of gendered expectations to gain access to the sector; and developing compensating perspectives, including material and relational justifications and positive male roles.

The second theme, ‘the migrant experience’, refers to a range of lived experiences associated with being a migrant, first and foremost. Migrant care workers often describe themselves in interviews as ‘resilient’, ‘committed’, or ‘determined’ and view these characteristics as assets and a positive contribution to social care (Hussein et al., 2011b). These characteristics are also evoked to cope with negative experiences many migrant care workers encounter. Discrimination, racism, and unfair treatment were often mentioned in the reviewed literature, especially for non-white migrant care workers (Hussein et al., 2011a, 2011b; Shutes & Walsh, 2012; Datta et al., 2010; McGregor, 2007; Stevens et al., 2012; Walsh & Shutes, 2013). Racism, however, is not necessarily open and direct; it is often expressed through cultural and linguistic ‘grievances’ as well as racialised assumptions/expectations about competence and character (Walsh & Shutes, 2013). Support from management and co-workers is crucial, but not always offered in the context of personalisation and user choice, where migrant care workers are often expected to tolerate racism, particularly from people seen as ‘vulnerable’ (Shutes & Walsh, 2012; Stevens et al., 2012). Many migrant care workers are part of global care chains, with families left behind and/or a lack of familial networks in the UK. In the context of restrictive migration policies, family expectations and managing their own caring arrangements can put pressure on migrants and increase their vulnerability to exploitation or limit their opportunities for advancement (Datta et al., 2010; McGregor, 2007).

The third theme ‘risks and vulnerabilities’ highlights that migrant care workers are more vulnerable to risks created by the structural combination of personalisation and care work organisation. These include precarity, isolation, emotional challenges, gendered risks of abuse, and unfair treatment (Shutes, 2012; Shutes & Walsh, 2012; Christensen & Manthorpe, 2016). The nature of home care, particularly the blurring between private and public, employer, and client aspects, can further complicate this problem in the context of a marketised system (Shutes & Walsh, 2012).

The fourth theme to emerge from this review is ‘relationships and values in care’. This is about how migrants see their role and contribution to social care. Several studies highlight that working in care—despite all the difficulties—is a valued experience for many migrant care workers and find that relationships with clients are a core determinant of positive outcomes (McGregor, 2007; Hussein et al., 2013). Relational aspects of care are seen as particularly important in home care (Walsh & Shutes, 2013), and many migrant workers construct work as ‘nurturing’ and ‘holistic’ and clients as ‘family’, placing emphasis on their own societal and cultural values, such as respect and care (McGregor, 2007; Datta et al., 2010; Hussein et al., 2013). This might result in tensions between personal values and the commodified and corporatised system of care, with implications for the emotional wellbeing of migrant care workers (Datta et al., 2010).

#### Service User Outcomes

Few studies explored the experiences of service users in relation to social care provided by migrant workers (Manthorpe et al., 2012a; Manthorpe et al., 2012b; Walsh & Shutes, 2013). Manthorpe et al. (2012b) identified four ‘ideal types’ in terms of satisfaction with care and preference for British care workers. While some service users voice openly racist and discriminatory views and preferences for care workers, many people are more cautious in expressing such views. Key concerns from the perspective of service users are cultural and linguistic proficiency and relationships with migrant care workers. While cultural and linguistic ‘grievances’ are often an expression of racism and discrimination, there is also evidence that a lack of cultural and linguistic proficiency—which makes communication ‘hard work’—may be associated with negative user outcomes, such as loneliness and isolation, especially for more vulnerable people (Manthorpe et al., 2012a). Characteristics associated with the ‘migrant experience’ such as resilience, commitment, and maturity, however, are often highly valued by service users and can to some extent compensate for a lack of cultural and linguistic proficiency that has the potential to improve with time and continuity (Manthorpe et al., 2012a; Walsh & Shutes, 2013).

#### Employer Outcomes

The three themes for employer outcomes are the following: migrant workers are a *source of flexibility*; impact on *quality of care*; and the *burden of employing* migrant care workers. Migrant care workers fill gaps in the supply of British-born workers and provide an important ‘source of flexibility’ for providers in three key aspects (Shutes & Chiatti, 2012): (1) migrant workers, especially initially, are more likely to accept less favourable conditions; (2) they are more ‘available’ for work (i.e. willing to work longer or unsocial hours); and (3) they are perceived to be more ‘committed’ and ‘reliable’ by their employers, most likely due to their immigration status or recent migration experience. As put by Hussein et al. (2011b), ‘the main reason why the English care sector recruits migrants is their willingness to do work that may be seen as unattractive by the local population. This appears to be more important than other attributes’ (p. 295).

The ‘burden and challenges’ theme highlights some of the difficulties associated with employing migrant workers: induction and training for new migrants; tensions arising from mediating racialised/discriminatory user preferences and workers’ rights to non-discrimination and dignity; and ‘paperwork’ (Cangiano & Shutes, 2010; Shutes & Walsh, 2012). Increasingly complex immigration rules and requirements put administrative and financial pressure on employers and create ‘risk work’ associated with ‘getting it wrong’, an issue that particularly affects smaller providers and may prevent some from employing job applicants subject to immigration control (Manthorpe et al., 2018). There are some suggestions of relatively high levels of irregular employment of migrant workers in social care (Cangiano & Shutes, 2010; Shutes, 2012); however, evidence is lacking in this area.

No studies were set out to specifically investigate the impact of employing migrant care workers on ‘quality of care’, but where this is discussed, provider organisations do not report any negative impact and some note improvements (Walsh & Shutes, 2013).

#### Workforce Outcomes

Only two studies commented on workforce outcomes and highlighted that migrant care workers are ‘structurally different’ from the general social care workforce. Firstly, they are ‘structurally positioned’ in low-paid and precarious jobs, especially in the private sector (Shutes & Chiatti, 2012). Secondly, recent migrant workers are more likely to be employed in ‘direct care’ roles, more likely to be working full time, and they are better qualified yet less likely to hold managerial or supervisory positions; they are younger, and a higher proportion are male compared to the social care workforce as a whole (Hussein et al., 2011c).

#### Sustainability

We considered the factors that would allow the supply and demand for migrant care workers to be maintained at functional (i.e. existing) levels. On the supply side, two key themes were identified. First is the ‘role of perception’ in destination country and sector choice. Migrants exercise a degree of agency in their decisions, and thus, the perceived accessibility and attraction of a country may be as important as its immigration policies (Christensen et al., 2017). Migration to the UK is aided by historical ties, existing migrant networks, and pragmatism, such as acquisition of English language. The availability of jobs in social care that do not require formal qualifications is recognised by migrants and acts as a ‘pull’ factor for those seeking to gain a foothold in the local labour market (Hussein et al., 2013).

Second, social care taps into the *stock of migrants* with right to work already in the country, and only a minority of migrant care workers were recruited directly from outside the UK (Cangiano & Shutes, 2010). Fluctuations and a fall in the number of migrants in the context of Brexit create uncertainties and challenges for employers particularly in home care and certain areas of the country (Read & Fenge, 2019). Although there is increased interest in employing refugees in social care, this is unlikely to be sufficient to fill gaps in workforce supply (Hussein et al., 2011a).

On the demand side, ‘structural factors’ and *immigration rules* shape demand for migrant workers. The relative importance of migrant care workers has increased within an expanding sector in the recent decade. Due to the uneven distribution of migrant workers, some regions and sectors are likely to be more vulnerable to a sharp drop in the supply of migrant workforce than others (Cangiano & Shutes, 2010). Demand for migrant care workers can be negatively affected by complex immigration rules and the requirement for employers to enforce immigration regulations, which can make it financially unfeasible and unsustainable for smaller employers to hire migrant workers, as highlighted by provider experiences after the 2012 visa reforms (Manthorpe et al., 2018).

One report (Independent Age, 2016) examines the combination of supply and demand side factors to estimate the workforce gap in social care by 2037. Taking the current ratio of care workers to older people taken as a baseline, it was projected that demand would outstrip the supply of social care workers, with migrant care workers needed to fill gaps. The report shows that zero net migration could leave a shortfall of up to 400,000 workers in the sector.

## Discussion

Migrant care workers provide more than a flexible source of labour to fill gaps in social care provision in the UK; they contribute to the sector in several ways, as highlighted by the findings of this scoping review. There has been a significant emphasis in the reviewed literature on ‘wellbeing failures’ (McGregor, 2014) experienced by migrant care workers, as well as migrant agency and experiences. Many of the findings in the reviewed studies resonate with the international literature on migrant care workers (e.g. Doyle & Timonen, 2010; Bauer & Österle, 2013; Di Rosa et al., 2012; Da Roit & Van Bochove, 2017; Theobald, 2017).

The review identified some gaps and limitations of existing UK research, particularly in the context of Brexit and changing immigration policies. To date, there has been less focus on what fosters positive wellbeing outcomes of migrant care workers and how migrant agency is mediated by immigration status (e.g. those with full right to work and those without, EU nationals, and non-EU nationals), race, class, and gender in the UK literature (Schwiter et al., 2018; Hamilton et al., 2019).

Although cultural and linguistic ‘grievances’ as well as racialised assumptions about competence and character have been highlighted in the reviewed literature, there has been limited research on sociocultural and psychological adaptation and socialisation—whereby migrant care workers adopt the rules and patterns of the receiving country (Ho & Chiang, 2015). The outcomes of these directly influence migrant care workers’ ability to provide ‘culturally congruent’ care (p. 239), as well as psychological wellbeing, and as a result affect the quality of care provided.

Racism towards migrant workers has received some attention in the literature, but the focus to date has been on ‘visible’ minorities. Eastern European migrants, even though they are predominantly white, have been described as a ‘racialised’ minority in the UK (Fox et al., 2012, 2015). Yet, their experiences in social care are not well explored or understood, especially in the context of Brexit (Kilkey & Ryan, 2020; Sotkasiira & Gawlewicz, 2020). There is limited research on the perception and lived experiences of discrimination among migrant care workers, including employer and wage discrimination.

Few studies focused on home care or live-in care, or compared different social care sectors, even though they are associated with different risks and challenges (Charlesworth & Howe, 2018). There is limited knowledge on how demand for, recruitment, and ‘matching’ of live-in migrant care workers to the needs or preferences of older care users or their families are shaped by their (real or assumed) attributes in the context of privately purchased care and the role of labour market intermediaries (Leiber et al., 2019; van Bochove & zur Kleinsmiede, 2020).

Social care has several distinguishing features that set it apart from other low-paid sectors with a high proportion of migrants (e.g. food processing, retail, and hospitality). Van Hooren et al. (2019) highlight three points: first, “migrants directly enter the sphere of the welfare state”; second, it is a “distinctly gendered segment of labour migration, not only because many of the migrants involved are women but also because the politics of care is often strongly gendered” (see also Hayes, 2018); and third, much migrant care work “takes place within the intimate sphere of the home”, which can create a demand for undocumented migration even in countries where this is relatively limited (p. 364). The UK social care sector is already vulnerable to risks of exploitation and modern slavery due to its purchaser-provider split and the use of complex tendering (commissioning) practices with weak oversight over labour supply chains (Emberson & Trautrims, 2019). The post-Brexit immigration system—the lack of legal work migration route for care work—is likely to add to existing pressures.

Finally, there is limited information on wellbeing outcomes for people who are supported by migrant care workers, although there is some suggestion in the literature that any negative outcomes may be outweighed or overcome by positive contribution of care workers’ experience and resilience. A better understanding of this, alongside future demand from an increasingly diverse ageing population, could provide an important contribution to any discussion on the future shape of care migration.

## Conclusion

In the current UK context, migrant care workers combine the risks and vulnerabilities of migrant status with the pressures of an already marginalised social care sector (Hussein, Manthorpe & Stevens 2011; Cunningham & James, 2014). With a growing and increasingly diverse ageing population, it is likely that the UK home care sector will continue to need migrant care workers to fill gaps in the workforce. The end of free movement of EEA workers and the new post-Brexit immigration system will create new risks and challenges that will be unfolding in the context of the COVID-19 pandemic. While the new immigration system is expected to reduce the labour supply by restricting migration into social care, the pandemic and its broader economic impact—the collapse of vacancies and job losses in other low-paid sectors (i.e. retail and hospitality)—has increased (at least temporarily) the labour supply in the care sector. Furthermore, the high rates of COVID-19 mortality in UK care homes (ONS, 2020a, 2020b) might turn people in need of care and support to sectors perceived as ‘safer options’, particularly live-in care that traditionally relies on migrants. Combined with the introduction of a more restrictive immigration system, this could create incentives for the emergence of an unregulated and informal ‘grey’ market of care in people’s homes.

## Footnotes

1 https://www.conservatives.com/our-commitments/taking-back-control-of-our-borders

## References

[CR1] Anderson A (2012). Europe’s care regimes and the role of migrant care workers within them. Journal of population ageing.

[CR2] Anttonen, A., Baldock, J., & Sipilä, J. (2003). *The young, the old, and the state: Social care systems in five industrial nations* (p. 13). Edward Elgar Publishing.

[CR3] Arksey H, O'Malley L (2005). Scoping studies: Towards a methodological framework. International journal of social research methodology.

[CR4] Bauer G, Österle A (2013). Migrant care labour: The commodification and redistribution of care and emotional work. Social Policy and Society.

[CR5] Bolton, J. & Townson, J. (2018). *Messages on the future of domiciliary care services.* Oxford: Institute of Public Care. Available: https://ipc.brookes.ac.uk/publications/Messages-on-the-future-of-domiciliary-care-services.html (last accessed: 29/08/2020)

[CR6] Cangiano A (2014). Elder care and migrant labor in Europe: A demographic outlook. Population and Development Review.

[CR7] Cangiano A, Shutes I (2010). Ageing, demand for care and the role of migrant care workers in the UK. Journal of Population Ageing.

[CR8] Cangiano, A., Shutes, I., Spencer, S., & Leeson, G. (2009). *Migrant care workers in ageing societies: Research findings in the United Kingdom*. COMPAS, University of Oxford.

[CR9] Charlesworth S, Howe J (2018). The enforcement of employment standards in Australia: Successes and challenges in aged care. International Journal of Comparative Labour Law and Industrial Relations.

[CR10] Christensen K (2017). Life trajectories of migrant care workers in the long-term care sectors in Norway and the UK. Social Policy and Society.

[CR11] Christensen K, Manthorpe J (2016). Personalised risk: New risk encounters facing migrant care workers. Health, Risk & Society.

[CR12] Christensen K, Hussein S, Ismail M (2017). Migrants’ decision-process shaping work destination choice: The case of long-term care work in the United Kingdom and Norway. European Journal of Ageing.

[CR13] Conservative and Unionist Party. (2019). *Get Brexit done. Unleash Britain’s Potential.* Available: https://www.conservatives.com/our-plan

[CR14] Cunningham I, James P (2014). Public service outsourcing and its employment implications in an era of austerity: The case of British social care. Competition & Change.

[CR15] Da Roit B, Van Bochove M (2017). Migrant care work going Dutch? The emergence of a live-in migrant care market and the restructuring of the Dutch long-term care system. Social Policy & Administration.

[CR16] Da Roit B, Weicht B (2013). Migrant care work and care, migration and employment regimes: A fuzzy-set analysis. Journal of European Social Policy.

[CR17] Datta K, McIlwaine C, Evans Y, Herbert J, May J, Wills J (2010). A migrant ethic of care? Negotiating care and caring among migrant workers in London's low-pay economy. Feminist Review.

[CR18] Denton MA, Zeytinoğlu IU, Davies S (2002). Working in clients' homes: The impact on the mental health and well-being of visiting home care workers. Home health care services quarterly.

[CR19] Di Rosa M, Melchiorre MG, Lucchetti M, Lamura G (2012). The impact of migrant work in the elder care sector: Recent trends and empirical evidence in Italy. European Journal of Social Work.

[CR20] Doyle M, Timonen V (2010). Obligations, ambitions, calculations: Migrant care workers' negotiation of work, career, and family responsibilities. Social Politics.

[CR21] Dromey, J., & Hochlaf, D. (2018). *Fair care: A workforce strategy for social care*. IPPR.

[CR22] Emberson, C., & Trautrims, A. (2019). Public procurement and modern slavery risks in the English adult social care sector. In O. Martin-Ortega & C. M. O’Brien (Eds.), *Public Procurement and Human Rights: Opportunities, risks and dilemmas for the state as a buyer*. Edward Elgar Publishing. 10.4337/9781788116312.00021.

[CR23] Fox JE, Moroşanu L, Szilassy E (2012). The racialization of the new European migration to the UK. Sociology.

[CR24] Fox JE, Moroşanu L, Szilassy E (2015). Denying discrimination: Status, ‘race’, and the whitening of Britain's new Europeans. Journal of Ethnic and Migration Studies.

[CR25] Fries-Tersch, E., Tugran, T., Rossi, L., & Bradley, H. (2018). 2017 *Annual Report on Intra-EU Labour Mobility*. *Brussels, European Commission.*10.2767/974797.

[CR26] Hall, P., Needham, C., & Hamblin, K. (2020). Social care. In *Handbook on Society and Social Policy*. Edward Elgar Publishing.

[CR27] Hamilton, M., Hill, E., & Adamson, E. (2019). A ‘career shift’? Bounded agency in migrant employment pathways in the aged care and early childhood education and care sectors in Australia. *Journal of Ethnic and Migration Studies*, 1–21.

[CR28] Hayes, L. (2018). *The crisis in social care is connected to the gendered inadequacy of labour law. British Politics and Policy at LSE* (24 Apr 2018). Blog Entry. https://blogs.lse.ac.uk/politicsandpolicy/gender-in-social-care-and-labour-law/ (accessed: 10/06/2019)

[CR29] Ho KH, Chiang VC (2015). A meta-ethnography of the acculturation and socialization experiences of migrant care workers. Journal of advanced nursing.

[CR30] Home Office. (2020). *Policy paper: The UK's points-based immigration system: Policy statement.* Available: https://www.gov.uk/government/publications/the-uks-points-based-immigration-system-policy-statement/the-uks-points-based-immigration-system-policy-statement#the-uks-points-based-system

[CR31] Hudson B (2019). Commissioning for change: A new model for commissioning adult social care in England. Critical Social Policy.

[CR32] Hussein S (2018). Job demand, control and unresolved stress within the emotional work of long term care in England. International Journal of Care and Caring.

[CR33] Hussein S, Christensen K (2017). Migration, gender and low-paid work: on migrant men’s entry dynamics into the feminised social care work in the UK. Journal of Ethnic and Migration Studies.

[CR34] Hussein S, Stevens M, Manthorpe J (2011). What drives the recruitment of migrant workers to work in social care in England?. Social Policy and Society.

[CR35] Hussein S, Manthorpe J, Stevens M (2011). Exploring the potential of refugees and asylum seekers for social care work in England: A qualitative study. Health & social care in the community.

[CR36] Hussein S, Manthorpe J, Stevens M (2011). The experiences of migrant social work and social care practitioners in the UK: Findings from an online survey. European Journal of Social Work.

[CR37] Hussein S, Manthorpe J, Stevens M (2011). Social care as first work experience in England: A secondary analysis of the profile of a national sample of migrant workers. Health & social care in the community.

[CR38] Hussein S, Stevens M, Manthorpe J (2013). Migrants’ motivations to work in the care sector: experiences from England within the context of EU enlargement. European journal of ageing.

[CR39] Hussein S, Ismail M, Manthorpe J (2016). Changes in turnover and vacancy rates of care workers in England from 2008 to 2010: panel analysis of national workforce data. Health & social care in the community.

[CR40] Independent Age. (2016). *Brexit and the future of migrants in the social care workforce*.

[CR41] Kilkey, M., & Ryan, L. (2020). Unsettling events: understanding migrants’ responses to geopolitical transformative episodes through a life-course lens. *International Migration Review* early on-line view.

[CR42] Leiber S, Matuszczyk K, Rossow V (2019). Private labor market intermediaries in the Europeanized live-in care market between Germany and Poland: A typology. Zeitschrift für Sozialreform.

[CR43] Lyon D, Glucksmann M (2008). Comparative configurations of care work across Europe. Sociology.

[CR44] MAC (Migration Advisory Committee). (2018). EEA migration in the UK: Final report. *London: Migration Advisory Committee* https://assets.publishing.service.gov.uk/government/uploads/system/uploads/attachment_data/file/741926/Final_EEA_report.PDF*.* (Accessed: 10/06/2019)

[CR45] Manthorpe J, Hussein S, Stevens M (2012). Communication with migrant workers: The perspectives of people using care services in England. Practice.

[CR46] Manthorpe J, Hussein S, Stevens M, Moriarty J (2012). User and carer experiences of international social care workers in England: Listening to their accounts of choice and control. Australian Social Work.

[CR47] Manthorpe J, Harris J, Stevens M, Moriarty J (2018). ‘We’re effectively becoming immigration officers’: Social care managers’ experiences of the risk work of employing migrant care workers. Health, Risk & Society.

[CR48] McGregor J (2007). ‘Joining the BBC (British Bottom Cleaners)’: Zimbabwean migrants and the UK care industry. Journal of ethnic and migration studies.

[CR49] McGregor, J. A. (2014). Chapter 14: Human wellbeing and sustainability: interdependent and intertwined. In G. Atkinson, S. Dietz, E. Neumayer, & M. Agarwala (Eds.), *2014. Handbook of sustainable development* (pp. 217–234). Edward Elgar Publishing.

[CR50] Morris M (2020). *Building a post-Brexit immigration system for the economic recovery*.

[CR51] NAO. (2018). *Adult social care at a glance.* London: National Audit Office. Available: https://www.nao.org.uk/wp-content/uploads/2018/07/Adult-social-care-at-a-glance.pdf (accessed on 10/06/2019)

[CR52] Needham C (2011). Personalization: From story-line to practice. Social policy & administration.

[CR53] ONS. (2020a). Deaths involving COVID-19 in the care sector, England and Wales: Deaths occurring up to 12 June 2020 and registered up to 20 June 2020 (provisional). Office for National Statistics. Available: https://www.ons.gov.uk/peoplepopulationandcommunity/birthsdeathsandmarriages/deaths/articles/deathsinvolvingcovid19inthecaresectorenglandandwales/deathsoccurringupto12june2020andregisteredupto20june2020provisional (accessed: 29/08/2020)

[CR54] ONS. (2020b). EMP06: Employment by country of birth and nationality. Availability: https://www.ons.gov.uk/employmentandlabourmarket/peopleinwork/employmentandemployeetypes/datasets/employmentbycountryofbirthandnationalityemp06 (accessed 01/12/2020)

[CR55] Owens J, Mladenov T, Cribb A (2017). What justice, what autonomy? The ethical constraints upon personalisation. Ethics and Social Welfare.

[CR56] Pollard, N., Latorre, M., & Sriskandarajah, D. (2008). Floodgates or turnstiles. *Post-EU Enlargement Migration Flows to (and from) the UK*.

[CR57] Ravalier J, Morton R, Russell L, Rei Fidalgo A (2019). Zero-hour contracts and stress in UK domiciliary care workers. Health & social care in the community.

[CR58] Read R, Fenge LA (2019). What does Brexit mean for the UK social care workforce? Perspectives from the recruitment and retention frontline. Health & social care in the community.

[CR59] Schwiter K, Strauss K, England K (2018). At home with the boss: Migrant live-in caregivers, social reproduction and constrained agency in the UK, Canada, Austria and Switzerland. Transactions of the Institute of British Geographers.

[CR60] Shutes I (2012). The employment of migrant workers in long-term care: Dynamics of choice and control. Journal of Social Policy.

[CR61] Shutes I, Chiatti C (2012). Migrant labour and the marketisation of care for older people: The employment of migrant care workers by families and service providers. Journal of European social policy.

[CR62] Shutes I, Walsh K (2012). Negotiating user preferences, discrimination, and demand for migrant labour in long-term care. Social politics.

[CR63] Simonazzi A (2008). Care regimes and national employment models. Cambridge Journal of Economics.

[CR64] Simpson JM, Esmail A, Kalra VS, Snow SJ (2010). Writing migrants back into NHS history: Addressing a ‘collective amnesia’and its policy implications. Journal of the Royal Society of Medicine.

[CR65] Skills for Care. (2020). *Individual employers and the personal assistant workforce report, 2020.* Leeds: Skills for Care, Available at www.skillsforcare.org.uk/topics (last accessed: 29/08/2020)

[CR66] Sotkasiira, T., & Gawlewicz, A. (2020). The politics of embedding and the right to remain in post-Brexit Britain. *Ethnicities, early on-line view.*

[CR67] Stevens M, Hussein S, Manthorpe J (2012). Experiences of racism and discrimination among migrant care workers in England: findings from a mixed-methods research project. Ethnic and Racial Studies.

[CR68] Theobald H (2017). Care workers with migration backgrounds in formal care services in Germany: A multi-level intersectional analysis. International Journal of Care and Caring.

[CR69] Thomas, J., Harden, A., & Newman, M. (2012). Synthesis: Combining results systematically and appropriately. In D. Gough, S. Oliver, & J. Thomas (Eds.), *An Introduction to Systematic Reviews* (pp. 179–227). Sage Publications.

[CR70] van Bochove M, zur Kleinsmiede D (2020). Broadening the scope of live-in migrant care research: How care networks shape the experience of precarious work. Health & Social Care in the Community.

[CR71] Van Hooren FJ (2012). Varieties of migrant care work: Comparing patterns of migrant labour in social care. Journal of European Social Policy.

[CR72] Van Hooren, F., Apitzsch, B., & Ledoux, C. (2019). The politics of care work and migration. In A. Weinar, S. Bonjour, & L. Zhyznomirska (Eds.), *The Routledge Handbook of the Politics of Migration in Europe* (pp. 363–373). Routledge. 10.4324/9781315512853-34.

[CR73] Walsh K, Shutes I (2013). Care relationships, quality of care and migrant workers caring for older people. Ageing & Society.

[CR74] Williams F (2012). Converging variations in migrant care work in Europe. Journal of European Social Policy.

